# Total elbow arthroplasty in elderly trauma patients: adding a new perspective for functional evaluation

**DOI:** 10.1007/s00068-022-01921-2

**Published:** 2022-03-05

**Authors:** Nils Mühlenfeld, Ingo Marzi, Johannes Frank

**Affiliations:** Department of Trauma, Hand and Reconstructive Surgery, University Hospital Frankfurt, Theodor-Stern-Kai 7, 60590 Frankfurt/Main, Germany

**Keywords:** Total elbow arthroplasty, Purdue Pegboard, Elderly trauma patient, Dexterity, Complex elbow fractures, Follow-ups

## Abstract

**Purpose:**

Total elbow arthroplasty (TEA) has evolved over the last years, with satisfactory early results, mainly not only in degenerative arthritis, but also increasingly after trauma. Outcome studies in recently published papers are mainly based on the range of motion (ROM), complication rate as well as patient-reported outcome scales and questionnaires. The purpose of this study was to add a new perspective with the “Purdue Pegboard” skill tests in a homogenous set of elderly trauma patients to contribute to a more precise objective outcome measurement in this specific population.

**Methods:**

A retrospective review was performed on a consecutive cohort of all patients with age above 60 years that received TEA after trauma. Data from follow-up examinations over a standardized time-schedule within 2 years after TEA were included. Mayo Elbow Performance Score (MEPS), “Disability of Arm, Shoulder and Hand” (DASH) Questionnaire, ROM as well as test-scores using the Pegboard test were evaluated.

**Results:**

Mean age was 76.0 years ± 10.3. Indications for TEA were posttraumatic arthrosis in 68.8% (*n* = 11) and extensive fractures that could not be reconstructed surgically in 31.3% (*n* = 5). The mean score of MEPS was 82.81 ± 16.63 and 29.18 ± 12.01 in the DASH. ROM presented with a mean of 109.7° ± 15.4. Patients demonstrated good, but marginally reduced test scores in the Pegboard skill tests in comparison with the healthy reference population. No relevant differences between the arm with and the arm without TEA (0.3 ± 3.6; *p* = 0.715) were noted after 2 years.

**Conclusion:**

In the elderly trauma patient with complex fractures of the elbow, TEA is a good alternative to joint reconstruction using various osteosynthesis techniques. TEA is able to avoid revision surgery after open reduction and internal fixation of complex fractures. In cases of failed reconstruction, it is also a viable secondary procedure in posttraumatic arthrosis. Good outcomes in functionality and dexterity can be achieved. Skill tests like the Purdue Pegboard could add a valuable perspective in assessing functional outcomes after TEA.

## Introduction

Total elbow arthroplasty (TEA) has evolved over the last years. Indications usually include advanced rheumatoid arthritis, post-traumatic arthrosis and acute fractures in the elderly patient which could not be reconstructed with a reasonable outcome, mostly after failed conservative treatment or complex distal humerus fractures [1–8].

Current literature with relatively small sample sizes report satisfactory outcomes for the use of TEA in older patients in the short term [1, 3, 9–12]. However they are mainly based on range of motion (ROM) and patient-oriented questionnaires that focus on activities of daily life, like the Mayo Elbow Performance Score (MEPS) [13] and the “Disability of Arm, Shoulder and Hand” (DASH) Questionnaire [14], which take an increasingly prominent role in assessing elbow function [13, 15, 16]. Standardized and validated performance tests with instruments that measure dexterity are rarely performed after TEA. Therefore, little is known about the objectively measurable skill level. Especially in older patients, often limited in their functional ability related to daily activities and mobilization [17–21] such measurement could add a promising new perspective for objective assessments.

The purpose of this study was to supplement the clinical outcome, MEPS and DASH with a new perspective resulting from the “Purdue Pegboard” skill test [17, 18] in a homogenous set of trauma patients older than 59 years, at a Level-I trauma center. Follow-up examinations were performed in a standardized time-schedule over the course of 2 years after TEA. Additionally, the epidemiology of TEA and its complications were evaluated.

## Materials and methods

### Study design and setting

Approval from the institutional review board and ethics committee of the Goethe University medical faculty (20-990) was obtained prior to performing this retrospective study. The study followed the STROBE guidelines for observational studies (Strengthening the Reporting of Observational Studies in Epidemiology) and the RECORD guidelines (Reporting of studies Conducted using Observational Routinely collected Data) [19, 20]. A retrospective review was performed on a consecutive cohort of all patients with age above 60 years that received TEA at the authors’ institution between 01/2010 and 10 /2020, to get a homogenous set of data. Exclusion criteria were missed follow-up examinations, pre-existing shoulder arthroplasty in either arm, as well as trauma to either arm within the examination period. Patients had prior been documented into a special register in our clinic after implantation. Patients’ characteristics and disease-specific aspects were transferred from the patient’s history to a digital database with baseline demographic variables including age and gender.

### Surgical procedure and rehabilitation protocol

The standard surgical procedure for TEA surgery was performed by a single senior surgeon in all patients. Linked modular Latitude total elbow arthroplasty (Tornier, USA) was used as an implant. Radial heads replacement components were used in nine patients, while the radial head was not replaced in seven patients.

After surgery long arm casts were applied. As soon as possible, depending on the postoperative swelling and pain level of the patient, elbows were treated in hinged elbow orthosis with protection against final pronation and supination but without limitation of flexion for 4 weeks. Movement exercises out of the cast were allowed after 3 weeks. After 4 weeks, the goal of therapy was to improve ROM. Increase of weight-bearing was allowed after a total period of 10 weeks. Weight-bearing over five kilograms as well as risk sports were not allowed for the remainder of the patients’ lives. A protective orthosis was recommended for a total of 12 weeks.

### Follow-up examinations

After TEA, patients received regular follow-up examinations in an outpatient setting. In this study, data from follow up examinations after 6 weeks (FU1), after 12–14 weeks (FU2), after 16–24 weeks (FU3), and after 48–52 weeks (FU4) were included. Patients underwent standard radiological diagnosis with x-rays as well as the thorough examination performed by experienced surgeons specialized in orthopaedic trauma care. Documented data included the ROM (extension deficit, maximum flexion angle, pronation angle and supination angle), the pain level and stability.

**Fig. 1:**
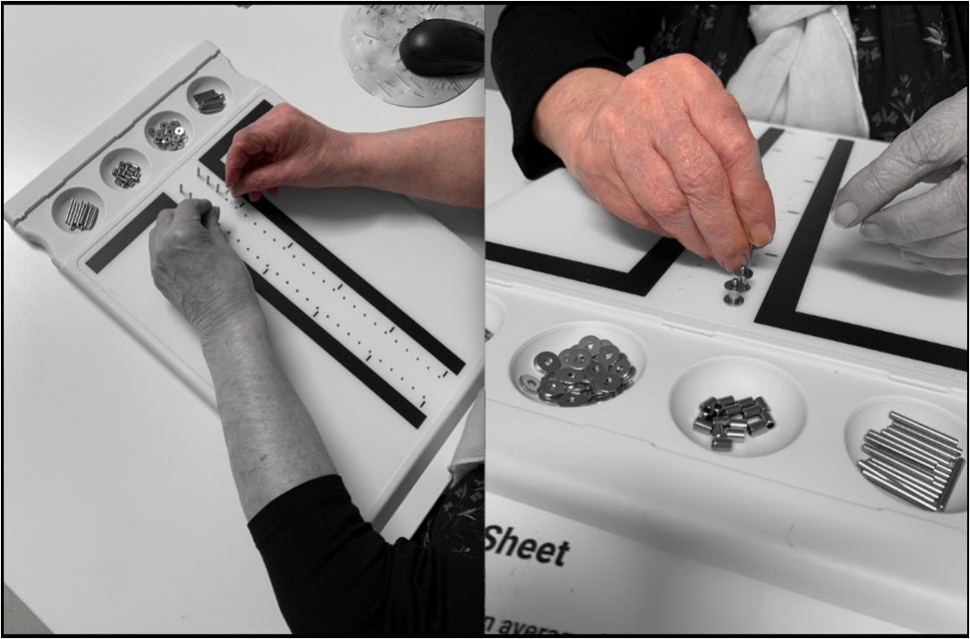
This figure shows a 76 year old female patient performing the “Purdue Pegboard” skill test [18]. In the left part the third subtest (“Both Arms”) is presented. Hereby the patient puts in as many rods as possible within 30 s, using both hands at the same time. The number of pairs is documented. In the right part the fourth subtest is presented (“Purdue Assembly”). Hereby the patient assembles as many structures as possible within 60 s, using both hands. The total number of parts is documented. The arm with TEA is presented in colour

In the final follow-up, designed for this study (FU5), after 2 years following TEA, patients’ Mayo Elbow Performance Score (MEPS) [13] and the “Disability of Arm, Shoulder and Hand” (DASH) Questionnaire [14] were documented. Additionally, all patients performed standardised dexterity tests with the “Purdue Pegboard”, which mainly measure coordinated motion of the hands, but also the arms as fine movements of the elbows and shoulders are necessary to perform the tasks (Lafayette Instrument, Indiana, USA) [17, 18, 21] (Fig. 1). With the Pegboard, four skill subtests were performed that are explained in detail:

- “Preferred Arm”: The patient puts in as many rods as possible within 30 s, using only the preferred hand. The total number of rods is documented.
- “Nonpreferred Arm”: The patient puts in as many rods as possible within 30 s, using only the nonpreferred hand. The total number of rods is documented.
- “Both Arms”: The patient puts in as many rods as possible within 30 s, using both hands at the same time. The number of pairs is documented.
- “Purdue Assembly”: The patient assembles as many “Purdue” structures as possible within 60 s, using both hands. The total number of parts is documented.

Each subtest was performed three times, the average out of the three trial scores is documented.

After FU5 the data set was inconclusive in some patients, which was due to patients passing away, non-traceable patients and patients that had not reached the timeline for the next follow up after 2 years. Therefore, no further follow-up examinations could be included in this study. To evaluate the complication rate, patient’s files were additionally scanned until the year 2020.

### Statistical analysis

For the (sub-)test score-values as well as the ROM-variables, the mean (M) and standard deviation (SD) as well as 95% Confidence Intervals (95% CI) were calculated across all individuals (*n* = 16). Because of the small subsets of male and female patients, we combined both sexes into one set of patients. Only patients with complete data for all follow-ups (FU1–FU5) were included. D'Agostino-Pearson normality test (“omnibus K2”) was used to determine if the data followed normal distribution. All the data followed normal distribution. A one-way ANOVA using the Geissner-Greenhouse correction was used for the ROM-values in every follow up to compare the improvements over the course of the rehabilitation period. Bonferroni corrections were used as statistical hypothesis testing for multiple comparisons. To compare the differences in scores in the Pegboard skill tests, a paired *t* test was used. Tests were calculated two-tailed using a 95% confidence level. *P* values < 0.05 were considered significant. All statistical analysis was performed using Prism Graphpad 8 (Graphpad Software, CA, San Diego).

## Results

### Epidemiology, indication and treatment

Data included 21 patients, 15 women (71.4%) and 6 men (28.6%), that received total elbow arthroplasty between 01/2010 and 10 /2020 in our department. Of the 21 patients, 5 were excluded from the data set. Three patients did not appear to all consecutive follow-ups (FU1-FU5), while two patients had passed away prior the final follow-up. Mean age of the included patients was 76.0 years ± 10.3 (range 60–96, 95% CI 71.0–81.0). In all but one patient, the right arm was the preferred arm and the left hand the nonpreferred arm.

Indications for TEA were posttraumatic arthrosis in 68.8% (*n* = 11) and extensive fractures that could not be reconstructed surgically in 31.3% (*n* = 5). Total elbow arthroplasty was performed in the right arm in 50.0% (*n* = 8) and the left arm in 50.0% (*n* = 8). The radial head was replaced in 37.5% (*n* = 6) and was not replaced in 62.5% (*n* = 10) where the defect could not be reconstructed.

### Range of Motion (ROM)

**Fig. 2:**
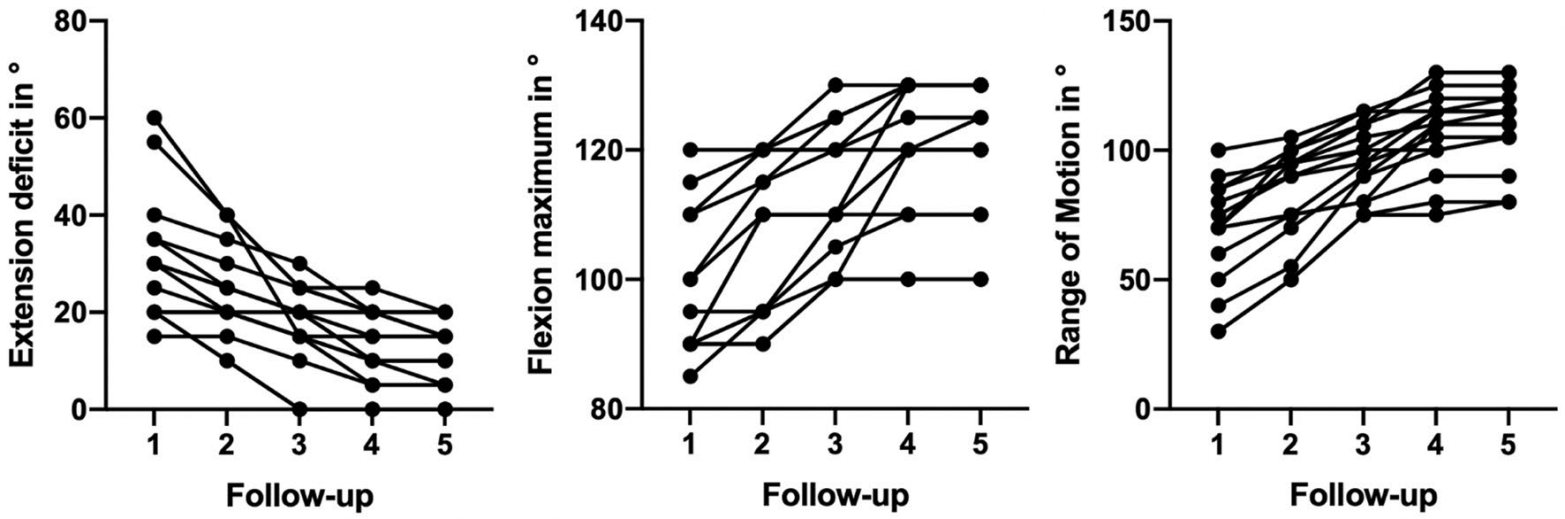
The progress of range of motion (ROM) over the course of the four follow ups is presented. Hereby the left picture shows the development of the angle of extension deficit. The middle picture shows the maximum flexion angle possible. The right picture demonstrates the progress in ROM between the extension deficit and maximum flexion angle. Within the first four follow-ups, a positive trend was noted, while after FU4, only three patients showed further improvement in the documented ROM

The development in ROM over the course of the five follow-ups is presented in Fig. 2. Significant improvements were documented between follow-ups for FU1-FU4 in the repeated measurements for the extension deficit (*p* < 0.001), maximum angle of elbow flexion (*p* < 0.001) as well as the total ROM between extension deficit and flexion angle (*p* < 0.001). From FU4 to FU5, only three patients demonstrated further improvement of the extension deficit (*p* = 0.041). Full extension of the elbow joint was only possible in one patient after 2 years. At the final follow-up mean extension deficit angle was 11.6° ± 7.0 (95% CI 7.8–15.3), mean flexion angle was 121.3° ± 10.7 (115.5–127.0) and mean ROM was 109.7° ± 15.4 (101.5–117.9). In FU5 mean pronation was 63.1° ± 22.4 (51.2–75.0) and mean supination was 55.0° ± 29.6 (39.2–70.8).

### Mayo elbow performance score and DASH-scores

**Fig. 3:**
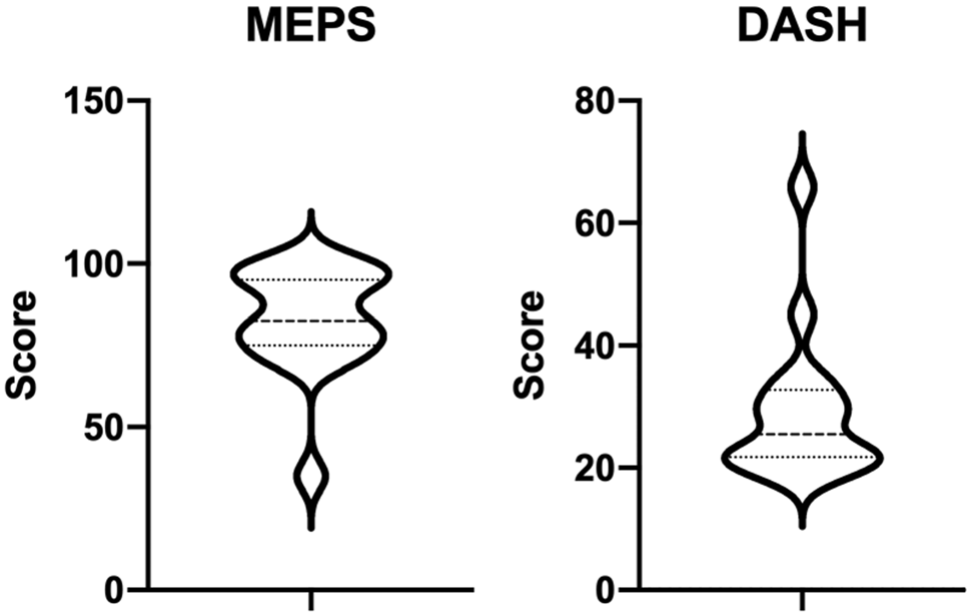
Data from the Mayo Elbow Performance Score (MEPS) (left) and the DASH (right) are presented in form of violin plots. While the MEPS showed overall good results compared to recent data, score-values from the DASH were not as favorable, but age appropriate

Patients overall presented with good results in the MEPS with a mean value of 82.81 ± 16.63 (95% CI 73.95–91.67). The DASH reported a mean value of 29.18 ± 12.01 (22.78–35.58) (Fig. 3).

### Purdue Pegboard skill tests

The results from the “Purdue Pegboard” skill tests are presented in Table 1.

**Table 1:** Results presented as mean, SD and 95%CI for each subtest (three trials per subtest) in the “Purdue Pegboard” skill test as well as the reference values for female patients, aged 70–79

Purdue pegboard skill test	Mean ± SD (Reference)	Mean ± SD	95% CI
Preferred arm	13.5 ± 1.5	12.2 ± 3.7	10.3–14.2
Nonpreferred arm	12.7 ± 1.5	11.4 ± 3.1	9.8–13.1
Both arms	10.5 ± 1.2	9.7 ± 2.9	8.2–11.2
Purdue assembly	28.5 ± 5.0	22.3 ± 6.2	19.0–25.6
Preferred—nonpreferred	0.8 ± 1.3	0.8 ± 3.5	− 1.–2.7
Arm with TEA		11.7 ± 3.8	9.7–13.7
Arm without TEA		12.0 ± 3.1	10.4–13.6
Arm without—arm with tea		0.3 ± 3.6	− 1.6–2.2

The reference values are presented as combined means and SD of male and female patients [22]

**Fig. 4:**
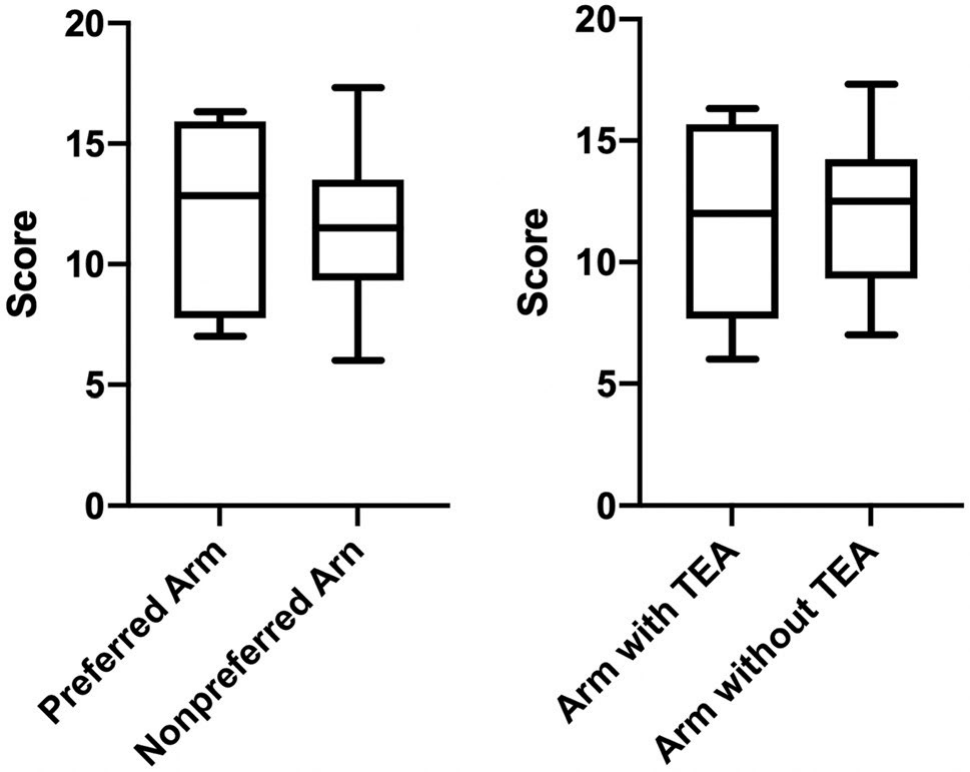
The results from the Purdue Pegboard dexterity tests are presented in form of box plots to compare the preferred hand with the nonpreferred hand (left) and the arm with TEA with the arm without TEA (right) (three trials per subtest)

Patients demonstrated reduced test scores compared to the reference population for the same age group. No statistically significant differences between the preferred arm and the nonpreferred arm (*p* = 0.378) was noted. Interestingly, there was also no statistical difference in the results between the arm with TEA and the arm without TEA (*p* = 0.715) (Fig. 4).

### Complications

All but one of the included patients returned to our apartment for irregular follow-ups after FU5. One patient suffered a periprosthetic fracture of the distal humerus after a fall, 6 years after TEA. It was treated with open reduction and internal fixation with a locking plate. TEA components did not have to be removed or reimplanted. In 93.8% (*n* = 15) of the patients no complication was documented.

## Discussion

The most important finding from this study is that elderly patients achieve good results in the Purdue Pegboard skill tests 2 years after TEA. However, the mean scores were reduced in comparison to the reference collective of the same age group. Yet this was only marginal. No relevant differences were noted between the preferred and nonpreferred arm.

Primary goal of total elbow arthroplasty is the stable and painless function [12]. Scores that reflect these outcome variables are the MEPS, the DASH, as well as the ROM. Recent data demonstrated good short-term results [1, 3, 9–11, 22]. Concerning ROM, the data from this study demonstrates a continuous improvement regarding the extension deficit and maximal possible flexion angle in the elbow joint. Continuous improvement between the follow-up appointments until week 52 was noted, while only few patients in this study improved their ROM after one year postoperatively, with only one patient reaching the ability to fully extend the elbow. Overall values in the ROM regarding extension and flexion were comparable to already published studies [11, 22, 23].

The MEPS has been traditionally used as an elbow rating system [24]. However, it was recently pointed out, that because of the combination of subjective and objective measures, the MEPS as a non-validated elbow rating system can lead to an overestimation of results after TEA [23]. Because large differences in MEPS-scores between rheumatic and traumatic indications in TEA-patients have been reported, this study demonstrated overall good results in trauma patients. This is consistent with recent reports    [22, 25–27].

The DASH is a recommendable complementary questionnaire with well documented validity, excellent test–re-test reliability and response to changes [15, 28]. The results from this study do not achieve DASH scores observed by a recent work in 2019, that presented patients with a mean score of 24 ± 12 (range 8–43) and a mean age of 66 years [29]. Excellent scores were reported by a German Study in 2012, which presented a mean score of 8.43 (range 0–28) for patients with TEA (mean age = 65) [22]. Evidently, the patients from both studies were significantly younger than the collective in this study. Elderly patients often present a preexisting reduced functionality in daily activities [30–33]. An increase of the DASH-Score with rising  age has been reported [34], which explains the reduced scores in this study compared to the previously mentioned publications. Age seems to be a key factor, comparing patient-reported outcome scales after TEA.

To add a new, different perspective to the discussed scores, which are prone to errors due to the subjective perspective of the patient at the time of questioning, the Purdue Pegboard skill tests were performed in this study. Our results showed close values to the reference values for the same age in each subtest provided in the Purdue Pegboard manual [18]. After 2 years, a reduced, but age-appropriate dexterity in elderly patients with TEA was demonstrated.

Interestingly, comparing the differences between operated and the non-operated arm an excellent outcome could be documented. The overall reduced scores in comparison to the reference groups might not be a direct result of TEA, but cohort specific for the set of patients in this study.

Recent findings have demonstrated the high risk of mid- and long-term complications like periprosthetic fractures, infection as well as aseptic loosening [1, 35–37]. However, the treatment of elbow fractures, especially complex distal humeral fractures, remains a challenging problem in older patients due to an inferior clinical outcome [7, 8, 38–41]. TEA which aims to result in a painless arm with good functionality related to daily activities, therefore poses a good alternative for the elderly patient, compared to complex osteosynthesis with potential revision surgery. Our results demonstrate only marginally reduced test scores in the Purdue Pegboard compared to the reference values of elderly patients [18] and no relevant difference between the arm with and the arm without TEA. In the elderly trauma patient with moderate demands, TEA therefore provides an option to be considered in elderly patients with complex fractures of the elbow, especially the distal humerus, providing an early return of function and pain reduction, as well as definitive treatment [4–8].

### Limitations

This study has several limitations. Because of the desired homogenous data set of elderly patients, we could only include a small number of patients over many years (*n* = 16). Also, of concern is the potential bias caused by patients who did not attend follow ups, as well was potential observer bias. Additionally, we only presented retrospective data from follow-ups within 2 years after TEA without a comparable control group. Further studies with larger cases observed over a longer period of time as well as controlled prospective interventional studies comparing ORIF with TEA are necessary in the future.

## Conclusion

In the elderly trauma patient with complex fractures of the elbow, TEA is a good alternative as a viable treatment to avoid revision surgery after open reduction and internal fixation. Excellent outcomes in functionality and dexterity can be achieved. Skill tests like the Purdue Pegboard could add a valuable perspective when assessing functionality outcomes after TEA.

## Data Availability

Not applicable.
